# Loss of SPRY2 contributes to cancer-associated fibroblasts activation and promotes breast cancer development

**DOI:** 10.1186/s13058-023-01683-8

**Published:** 2023-07-28

**Authors:** Huijuan Dai, Wenting Xu, Lulu Wang, Xiao Li, Xiaonan Sheng, Lei Zhu, Ye Li, Xinrui Dong, Weihang Zhou, Chenyu Han, Yan Mao, Linli Yao

**Affiliations:** 1 State Key Laboratory of Systems Medicine for Cancer, Shanghai Cancer Institute, Ren Ji Hospital, School of Medicine, Shanghai Jiao Tong University, 800 Dongchuan Road, Shanghai, 200240 People’s Republic of China; 2 Department of Breast Surgery, Renji Hospital, School of Medicine, Shanghai Jiao Tong University, Shanghai, People’s Republic of China; 3 Department of Pathology, The International Peace Maternity and Child Health Hospital of China Welfare Institution, School of Medicine, Shanghai Jiao Tong University, 910 Hengshan Road, Shanghai, 200030 People’s Republic of China; 4 Department of Human Anatomy, School of Basic Medical Sciences, Capital Medical University, Beijing, People’s Republic of China; 5 Beijing Key Laboratory of Cancer Invasion and Metastasis Research, Beijing, People’s Republic of China; 6 Department of Obstetrics and Gynecology, Shanghai Sixth People’s Hospital Affiliated to Shanghai Jiao Tong University School of Medicine, Shanghai, People’s Republic of China; 7 Department of Endocrinology, Qingpu Branch of Zhongshan Hospital Affiliated to Fudan University, 1158 Gongyuan Road, Shanghai, 201700 People’s Republic of China; 8 Breast Disease Center, The Affiliated Hospital of Qingdao University, No. 59 Haier Road, Qingdao, 266003 Shandong People’s Republic of China

**Keywords:** Microenvironment, Fibroblast, LDHA, Glycolysis, Stemness

## Abstract

The communication between tumor cells and tumor microenvironment plays a critical role in cancer development. Cancer-associated fibroblasts (CAFs) are the major components of the tumor microenvironment and take part in breast cancer formation and progression. Here, by comparing the gene expression patterns in CAFs and normal fibroblasts, we found SPRY2 expression was significantly decreased in CAFs and decreased SPRY2 expression was correlated with worse prognosis in breast cancer patients. SPRY2 knockdown in fibroblasts promoted tumor growth and distant metastasis of breast cancer in mice. Loss of stromal SPRY2 expression promoted CAF activation dependent on glycolytic metabolism. Mechanically, SPRY2 suppressed Y10 phosphorylation of LDHA and LDHA activity by interfering with the interaction between LDHA and SRC. Functionally, SPRY2 knockdown in fibroblasts enhanced the stemness of tumor cell dependent on glycolysis in fibroblasts. Collectively, this work identified SPRY2 as a negative regulator of CAF activation, and SPRY2 in CAFs may potentially be therapeutically targeted in breast cancer treatment.

**Supplementary Information:**

The online version contains supplementary material available at 10.1186/s13058-023-01683-8.

## Introduction

The ‘seed and soil’ theory was first proposed more than a century, following which the role of tumor microenvironment (TME) in cancer development has received widespread attention in recent years [1]. Numerous studies have proved the interaction between TME and tumor cells and pointed out that TME might play a key role in tumor development [2]. Therefore, targeting the TME has appeared as a new cancer treatment paradigm in many solid tumors.

Cancer-associated fibroblasts (CAFs) are one of the most abundant stromal components in the TME. CAFs are differentiated from quiescent fibroblasts and are characterized with high expression of vimentin–α smooth muscle actin (αSMA), fibroblast activation protein (FAP) and fibroblast-specific protein 1 (FSP1) [3]. Numerous studies have demonstrated the prominent roles of CAFs in cancer pathogenesis [3]. CAFs produce and remodel extracellular matrix (ECM) components. ECM with increased stiffness and abnormal physical structure can promote tumor cell growth and metastatic dissemination [2, 4]. Besides, CAFs can interact with cancer cells or other stromal cells through the secretion of growth factors, cytokines, and chemokines to promote cancer progression [5–7]. CAFs are therefore conspicuous stromal targets in many solid tumors, which requires a thorough understanding of their activation mechanism during tumor development.

Sprouty family genes (SPRYs) were discovered by Hacohen et al. and were evolutionarily conserved inducible inhibitor of signaling by receptor tyrosine kinases [8, 9]. Four SPRY homologs including SPRY1-4 (SPRY1-4) have been identified in mammals and have been demonstrated to implicate in various developmental and physiological processes. SPRY mediates cross-talk among different signaling pathways and orchestrates a multilayered regulatory system in cellular response [10]. It has been well established that SPRY family genes are essential for the proper development and physiological function of many organs including the lung, the vascular system, the kidney mammary gland, bone, prostate, and genitourinary tract [11]. A variety of studies have revealed the dysregulation and dysfunction of SPRY genes in cancer development [9, 12–14]. SPRYs play various functions depending on the cancer type or the SPRY isoform. Down-regulation of SPRY1 and SPRY2 occurs in prostate, liver, lung, and breast cancers [15–17]. Loss of SPRY1 reduces the growth of cutaneous melanoma and suppression of SPRY1 inhibits triple-negative breast cancer malignancy [18, 19]. In contrast, SPRY2 functions as a putative oncogene in colorectal cancer [20]. Breast cancer is characterized by ECM deposition, remodeling, and cross-linking that drive fibrosis to stiffen the stroma [21]. The stiffened stroma promotes the malignancy of tumor by enhancing tumor cell survival, growth and migration and it also induces angiogenesis and compromises anti-tumor immunity [22]. We previously uncovered a system-wide phosphorylation network regulated by SPRY proteins, suggesting the potential role of SPRY in mammary gland microenvironment [23]. Therefore, the function of SPRY family genes in breast cancer microenvironment is worth to be explored.

In this study, we identified the downregulation of Sprouty family gene SPRY2 in CAFs of breast cancer. Here, we further sought to delineate the role and mechanisms of SPRY2 in CAFs of breast cancer. Our data shed light on the mechanism underlying the cross-talk of CAFs with tumor cells and provided evidence for stromal therapeutic targets in breast cancer.

## Materials and methods

### Clinical specimens

Tissue microarray (TMA) containing breast cancer specimens obtained from 132 cases of breast cancer patients was purchased from Shanghai Outdo Biotech Inc (HBreD136Su02). All clinical samples were collected with informed consent under the Health Insurance Portability and Accountability Act (HIPAA) approved protocols.

### Isolation of mice mammary gland fibroblasts

Mammary gland fibroblasts were isolated as previously reported. In brief, mouse mammary glands from eight-week-old female mice were minced and digested in collagenase solution (DMED/F12 supplied with 0.2 g/mL collagenase, 0.2 g/mL trypsin, 5 µg/mL insulin, 50 µg/mL gentamicin, 5% fetal bovine serum (v/v) for 30 min. Fatty layer was removed after centrifugation and the left samples were treated with DNase I. After that, the samples were subjected to 4–5 times of differential centrifugation at 450 × g. The supernatant was collected and then centrifuged at 600 × g for 10 min. Cell pellets were then resuspended in DMEM with 10% (v/v) FBS, 1 × penicillin and streptomycin and seeded in culture dishes. 30 min later, the cells were changed with fresh fibroblast medium.

### Cell culture and reagents

Murine mammary carcinoma cell line EO771 and 4T1 cells, and the isolated mammary gland fibroblasts were cultured in DMEM under an atmosphere consisting of 95% air and 5% CO2 at 37 °C. The EO771 and 4T1 cells were sub-cultured at a ratio of 1:6 and the mammary gland fibroblasts were sub-cultured at a ratio of 1:3. Cells were treated with 2-Deoxy-D-glucose (2-DG; 5 mM; #HY-13966; MCE), SRC inhibitor KX2-391(#HY-10340, 100 nM, MCE).

### Subcutaneous xenograft model of breast cancer

Five-week-old C57BL/6 female mice were kept on a 12-h day/night cycle and fed a normal chow diet. EO771 tumor cells were mixed with shNC, sh*Spry2*-1, or sh*Spry2*-2 mammary gland fibroblasts at a ratio of 1:3, with each recombinant comprised of 1 × 10^5^ EO771 cells and 3 × 10^5^ fibroblasts. The cell mixtures were polymerized with rat tail collagen and overlaid with growth medium at 37 °C overnight before implantation. For subcutaneous xenograft model, the cell/collagen mixture was injected subcutaneously in the lower back of the mice. Four weeks after injection, mice were sacrificed, the tumors were isolated and weighed.

### Orthotopic xenograft model of breast cancer

Female Nu/nu mice were purchased from Shanghai Shengchang Bio-tech Co., Ltd (Shanghai, China). All animal studies were approved by the Shanghai JiaoTong University Institutional Animal Care and Use Committee. 2 × 10^5^ 4T1 cells mixed with 6 × 10^5^ shNC or sh*Spry2* fibroblasts were resuspended in culture medium containing 50% Matrigel (BD Biosciences). The suspension was then orthotopically implanted by injection into the 4th left mammary fat pad. At day 7, 14 and 20 after transplantation, tumor formation in mice were monitored by bioluminescence signals using an in vivo imaging system instrument. The signals from resected organs were also monitored ae day 20 after cell sacrifice.

### Lung metastasis model of breast cancer

A mixture of 1 × 10^5^ EO771 and 3 × 10^5^ fibroblasts were injected into tail vein of C57BL/6 female mice. Five weeks later, the mice were perfused and lungs were isolated. The lungs were paraffin embedded and serial Sects. (4 μm) were processed with hematoxylin and eosin (H&E) staining. Quantitation of metastasis on H&E-stained slides of lung lobes were performed by pathologist in a blinded manner. Lung tumor burden was quantitated using ImageJ software (NIH Image).

### Immunohistochemistry

Immunohistochemistry (IHC) were performed routinely. Briefly, the paraffin-embedded slices (4 μm thickness) were deparaffinized in 100% xylene and rehydrated in descending ethanol series from 100 to 75% and then in water according to standard protocols. After blocking in BSA (10%), the sections were incubated with primary antibodies overnight at 4 °C. On the second day, the sections were incubated with HRP conjugated secondary antibodies for 1 h at room temperature. The sections were finally stained with DAB substrate and counterstained by hematoxylin. All the sections were photographed with a photo microscope (Carl Zeiss). Specific antibodies used for immunohistochemistry were as follows: Ki67 (1:100, Servicebio, #GB111499), CD44 (1:100, Servicebio, GB112054).

SPRY2 protein expression in TMA of breast cancer patients was stained using anti-SPRY2 antibody (1:100, Proteintech, #11383-1-AP) following routine procedures. Pathology scores were assessed based on protein expression intensity in elongated (spindle-shaped) cancer-associated fibroblasts (CAFs) or normal fibroblasts according to the following criteria in a blinded manner: strong expression: dark brown staining in > 60% of tumor cells, scored as “ +  +  + ”; moderate expression: brown staining in > 60% of tumor cells, scored as “ +  + ”; weak expression: less brown staining, scored as “ + ”; and absent: no appreciable staining in tumor cells, scored as “-”. A score of “-” or “ + ” was defined as low expression and a score of “ +  + ” or “ +  +  + was defined as high expression”. These scores were determined in a blinded manner.

### Immunofluorescence staining

For Immunofluorescence staining in tissue sections, paraffin sections (5 μm) of mice tumors were deparaffinized, rehydrated with graded ethanol, and then subjected to antigen retrieval. Sections were blocked with 10% donkey serum and then incubated with anti-mice α-SMA (GB13044), anti-mice CK18 (Servicebio, #GB13232-M), anti-rabbit CD44 (Servicebio, #GB112054), or SPRY2 primary antibodies overnight at 4 °C. At the second day, the sections were incubated with Cy3 or FITC -conjugated secondary antibodies for 1 h at room temperature and then stained with 4’,6-diamidino-2-phenylindole (DAPI). All images were captured using a confocal microscope.

### Seahorse analyses

The assays for extracellular acidification rate (ECAR) in fibroblasts were performed with the Seahorse XF96 Flux Analyzer (Seahorse Bioscience, Agilent) according to the manufacturer’s instructions. Briefly, shNC or sh*Spry2* fibroblasts were seeded in a XF96-well plate at a density of 1 × 10^4^ per well. The medium was replaced with assay media at 1 h before the assay. For the glycolytic stress test, 10 mM glucose, 1 μM oligomycin and 50 mM 2-deoxyglucose (2-DG) were injected to the wells. The measurements were normalized by total protein quantitation. Above experiments were performed in triplicate manner and repeated twice.

### Mammosphere -formation assays

For mammosphere-formation assays, 4T1 cells were plated in 24-well ultralow attachment plates with a total number of 2000 cells/well. Cells were cultured in serum-free DMEM/F12 medium supplemented with 20 ng/ml human epidermal growth factor (EGF), 10 ng/ml human basic fibroblast growth factor (bFGF), 2% B27 supplement and mixed with CM from fibroblast cells at 1:1. Medium were replenished every 3 days. After 7 days, the number and size of primary floating spheres were counted. Spheres were dissociated for secondary sphere expansion under the same conditions. The number of tumorspheres (diameter > 50 μm) in each well were evaluated using an inverted microscope by two researchers to reduce the counting bias. The number of mammospheres was determined from at least three independent experiments.

### Determination of LDHA enzyme activity

The LHDA enzyme activity was determined using Lactate Dehydrogenase (LDH) Activity Assay Kit (#BC0685, Solarbio) following the standard the protocol.

### Construction of lentivirus constructs and cell transfection

For *Spry2* overexpression, full-length cDNA encoding mouse *Spry2* was amplified and cloned into a pCDH vector. shRNA constructs against mice *Spry2* and scrambled sequences we have designed were constructed into pLKO.1. The sequences of shRNA against mice *Spry2* were as follows: sh*Spry2*-1 TTAAGGCAACCCTTGGCTG; sh*Spry2*-2 TAGCTGAATTGTAGCAAGC. Lentiviral particles were generated using a three-plasmid-system, including pPACKH1-GAG, pPACKH1-REV, and pVSV-G, and then packaging in 293 T cells with jetPRIME® following recommended protocols. Fibroblasts were transduced with recombinant lentivirus plus 6 μg/ml polybrene. Three days after infection, cells were treated with 5 μg/ml puromycin to eliminate the uninfected cell and the puromycin-resistant cells were yield. The overexpression or knockdown efficiency of SPRY2 was detected by qPCR and western blot.

### Glucose and lactate measurement

Mammary fibroblasts were seeded into 6-well plates (1 × 10^5^ cells per well) and allowed to attach overnight. Culture medium was changed to free DMEM with no FBS and cultured for 24 h. Then, cell culture medium supplemented with 5% FBS was added and the cells were incubated for an additional 24 h. Culture media was collected and filtered with 0.22 μm filters to measure glucose and lactate concentration. Glucose concentration in the medium was measured using glucose content assay kit (#BC2505, Solarbio). Glucose consumption was calculated by the original glucose concentration deduced the glucose concentration in the medium. Lactate production was measured by the lactate content assay kit (#BC2235, Solarbio). Total protein was extracted from the cell pellets and protein concentration was quantified for normalization of the results obtained above.

### Quantitative real-time PCR (qRT-PCR)

Total RNA was prepared from the cell samples using RNAiso Plus (Takara, #9108Q). Reverse transcriptase PCR was performed using the PrimeScript RT-PCR kit (Takara, #RR600A). Real-time PCR reactions were performed using SYBR® Premix Ex Taq™ on a 7500 Fast Real-Time PCR Systems according to the manufacturer’s protocol. Data were normalized to the expression of the control gene (β-actin) calculated with 2^(−ΔΔCt)^ method for each experiment. Data represent the mean ± SD of three independent experiments. The sequences of primer pairs used for quantitative RT-PCR are listed in Additional file 1: Table S1.

### Immunoprecipitation analysis

Fibroblasts transfected with vector (NC) or HA-*Spry2* plasmids were lysed using RIPA lysis buffer (#G2002, Servicebio) with 1 × Protease/Phosphatase inhibitor cocktail. The protein A/G Co-IP beads (ShareBio, #SB-PR001) were pre-cleaned and incubated with anti-rabbit LDHA (Proteintech, #19987-1-AP), anti HA (Proteintech, #66006-2-Ig) or control IgG for 60 min in TBST at room temperature. The beads were then incubated with 100 mg total protein with gentle shaking at room temperature for 60 min. The immunocomplexes were collected and resolved on SDS-PAGE gel and then transferred to NC membranes (Millipore) to proceed with western blot.

### Western blot

Western blot assay was performed according to standard procedures. Cell lysis was extracted using RIPA lysis buffer and protein concentration was measured. Cell lysates were separated by 6–12% SDS-PAGE gel electrophoresis and transferred to a nitrocellulose membrane. After blocking with 5% skimmed milk, the membrane was probed with one of the following primary antibodies at 4℃ overnight: SPRY2 (1:1000), LDHA (1:2000, Cell signaling, #2012), Phospho-LDHA (Tyr10) (1:1000, Immunoway, #YP1385), SRC (1:1000, Proteintech, #11097-1-AP), HA (1:1000, Proteintech, #66006-2-Ig), MCT1(1:1000, Immunoway, # YN0868), MCT4(1:1000, Immunoway, # YT6150). On the second day, the membrane was washed with TBST and then incubated with HRP conjugated second antibody for 1 h at room temperature. Signals were detected by Odyssey imaging system (LI-COR Biosciences, Lincoln, NE).

### 3D-coclutures and flow cytometry

For determining the influences of fibroblasts on 4T1 cell stemness, 4T1 cells and shNC or sh*Spry2* fibroblasts were co-cultured in Matrigel. After 7 days, the picture of colonies was captured and cells were dissociated and suspended in PBS supplemented with 0.5% BSA for staining with APC-conjugated anti-CD44 (#103011, BioLegend) and PE anti-mouse CD24 Antibody (#138503, BioLegend) antibodies for 15 min at 4 °C. The stained cells were analyzed by flow cytometry (FACSAria™ III, Becton Dickinson) and FlowJo v.10.4.2 was used for further analysis.

### Bioinformatic analysis

GEO datasets (GSE9014, GSE10797 and GSE90505) containing RNA-sequencing data of normal fibroblasts and cancer-associated fibroblast from breast cancer patients were adopted for analysis. GEO datasets were analyzed by R packages “GEOquery” and “limma”. The survival of breast cancer cohort was analyzed with K-M plotter (http://kmplot.com/analysis/index.php?p=background). KEGG and GO pathway enrichment was carried out with R package “clusterProfiler”, “DOSE” and “enrichplot”. GSEA pathway enrichment was carried out with software GSEA (version 4.0.1).

Single-cell-sequencing data analysis was performed by online tool (Tumor Immune Single-cell Hub 2, TISCH2 http://tisch.comp-genomics.org/home/). Single-cell-sequencing data of breast cancer dataset (GSE111229) were adopted for analysis. Single-cell data GSE111229 (FKPM) were downloaded from the GEO database and downstream processing was performed using Seurat v4.2.0.

### Statistics

Data are presented as the mean ± SD unless stated otherwise. All the experiments were replicated at least three times. Statistical calculations were performed using the GraphPad Prism 7 software. Survival time was analyzed using Kaplan–Meier method and the *P* value was calculated by log-rank test. The statistical significance of differences between groups was calculated using unpaired Student’s t test, Mann–Whitney U test and one-way analysis of variance (ANOVA). Differences were considered statistically significant at *P < 0.05, **P < 0.01 and ***P < 0.001.

## Results

### Low SPRY2 expression in CAFs of breast cancer is associated with poor prognosis

**Fig. 1:**
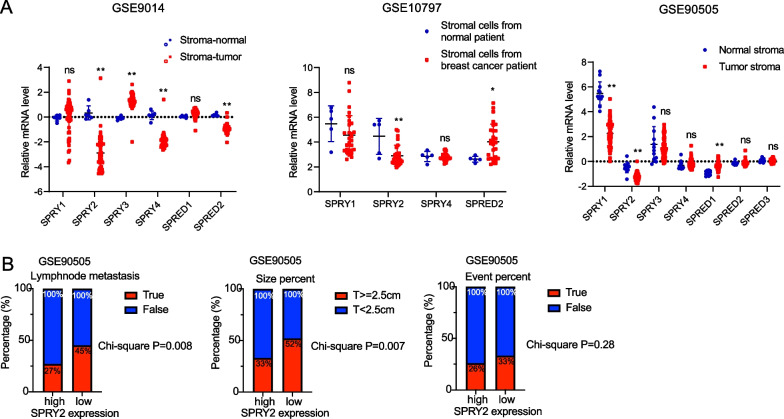
SPRY2 expression is decreased in fibroblasts of breast cancers. **A** SPRY/SPRED family in three Gene Expression Omnibus (GEO) datasets GSE9014, GSE10797 and GSE90505, containing expression profile data of CAFs and matching normal fibroblasts (NF) from breast cancer patients. **B** Correlation analysis of SPRY2 expression with lymphnode metastasis, tumor size and survival event percentage of patients with BRCA from GSE90505 (Fisher’s exact test). *ns* non-significant, *P < 0.05, **P < 0.01, ***P < 0.001

We first observe the mRNA expression pattern of SPRY/SPRED family genes in different cell types of breast cancer from human patients by analyzing single-cell sequencing data online (TISCH2, http://tisch.comp-genomics.org/home/). We found high mRNA expression of SPRY/SPRED genes in fibroblasts (Additional file 2: Figure S1). To identify the potential SPRY/SPRED family genes dysregulated in CAFs, we first analyzed the expression of SPRY/SPRED family genes in three Gene Expression Omnibus (GEO) datasets GSE9014, GSE10797 and GSE90505, which contain expression profile data of cancer-associated fibroblasts (CAFs) and matching normal fibroblasts (NFs) from breast cancer patients. We found that SPRY2 expression was uniformly decreased in CAFs compared with NFs in all three datasets (Fig. 1A). To test the predictive value of fibroblasts expressing SPRY2 in breast cancer prognosis, data derived from GSE90505 with clinical follow-up information were analyzed. We found low SPRY2 expression in CAFs predicted more lymphnode metastasis, bigger tumor size and higher frequency of survival event in patients with breast cancer (Fig. 1B).

**Fig. 2:**
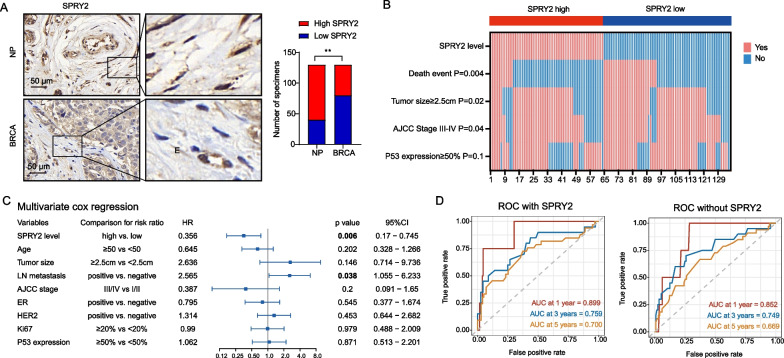
Decreased SPRY2 protein expression in stroma predicts a poor prognosis in patients with BRCA. **A** IHC staining of SPRY2 in a human breast cancer tissue microarray from 132 cases of breast cancer patients. Representative SPRY2 images were shown at left panel. The percentage of tissue cores displaying low and high staining in fibroblasts of normal breast and BRCA lesions was shown at right panel. Scale bar: 50 µm, **P < 0.01. **B** The correlation between SPRY2 expression in CAFs and patients’ death event, tumor size, AJCC stage and P53 expression was conducted based on the SPRY2 expression in CAFs from the IHC staining of the tissue microarray. **C** Multivariate Cox regression analysis of SPRY2 expression and clinicopathological factors for overall survival. *HR* Hazard ratio; *CI* Confidence interval. **D** ROC curves of multivariate cox model with or without SPRY2 expression in fibroblasts from IHC staining of human breast cancer tissue microarray

To further determine the protein expression of SPRY2 in fibroblasts of breast cancer, we performed immunohistochemical (IHC) analysis in a human breast cancer tissue microarray (TAM) from 132 cases of breast cancer patients. As a result, SPRY2 expression was highly expressed in stroma of normal breast, but the expression was decreased in the stroma of breast cancer (Fig. 2A). We next explored the prognostic value of SPRY2 protein and found the correlation between low SPRY2 expression in CAFs and patients’ higher frequency of survival event, larger tumor size and higher AJCC stage (Fig. 2B). Multivariate Cox regression analysis found low SPRY2 expression was one of the independent clinicopathological factors for poor overall survival in breast cancer patients (Fig. 2C). ROC curve further showed the predictive performance of cox model with SPRY2 and clinicopathological factors are higher than the cox model with only clinicopathological factors (Fig. 2D). These results suggest low expression of SPRY2 may predict poor prognosis in breast cancer patients.

### SPRY2 knockdown in CAFs increased tumor cells proliferation and invasion in mice

**Fig. 3:**
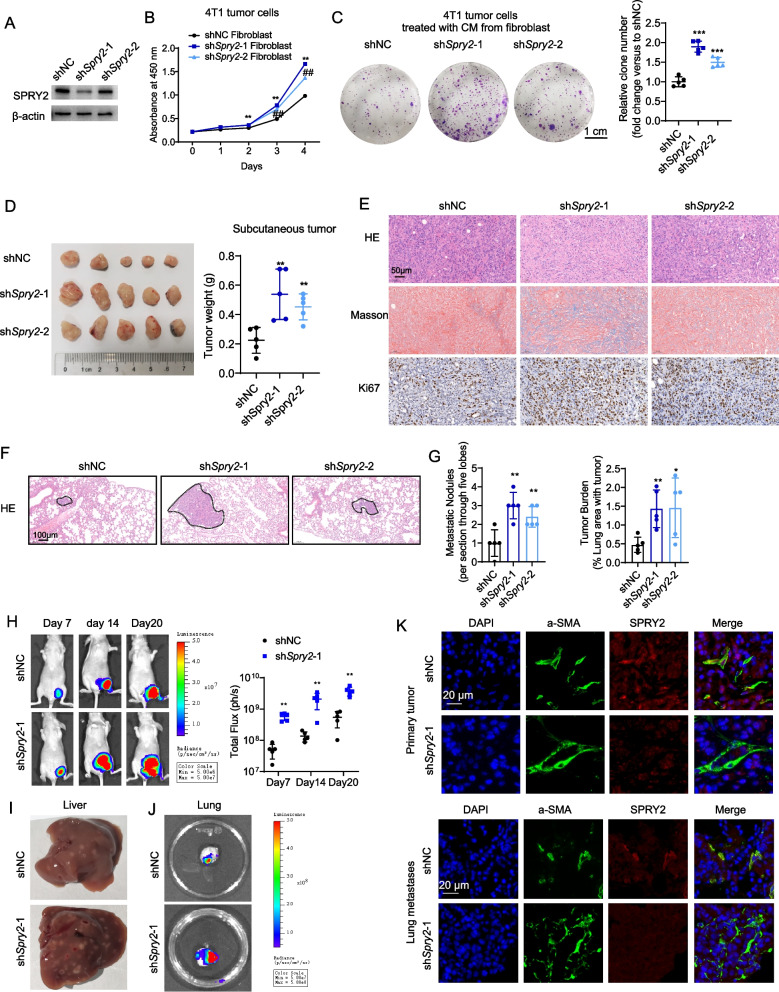
SPRY2 knockdown in stroma promotes breast cancer progression. **A** Western blot analysis showing the protein expression of SPRY2 in fibroblasts after shRNA mediated SPRY2 knockdown. **B** Quantification of 4T1 cells treated with conditioned media (CM) from control or sh*Spry2* fibroblasts by CCK8 assay. **C** Colony formation assay of 4T1 tumor cells treated with CM from control or sh*Spry2* fibroblasts. Scale bar: 1 cm. **D** Mice were subcutaneously implanted with EO771 cells in combination with shNC or sh*Spry2* fibroblasts. Representative tumors images from each group. Right graph showing the tumor weights from each group at the experimental endpoint. **E** Representative HE, Masson staining and immunohistochemical images of Ki67 staining from shNC and sh*Spry2* subcutaneous xenograft tissues. Scale bar:50 µm. **F**, **G** Mice were implanted with EO771 cells in combination with shNC or sh*Spry2* fibroblasts into tail vein and the lung was resected after 5 weeks. Representative HE staining images in sections of lung tissue in (**F**). Scale bar:100 µm. Graph showing the number of metastatic nodules and the percentage of lung area with tumor in the lung of mice in (**G**). Data are presented as mean ± SD. (*n* = 5 independent samples). **H** Luciferase tagged 4T1 cells mixed with shNC or sh*Spry2* fibroblasts were transplanted in the fourth left mammary fat pad of female nude mice. Tumor growth in the mammary fat pad was monitored using bioluminescence imaging on day 7, 14 and 20 (image scales in p/s/cm^2^/sr, *n* = 5 mice). The right graph showing the corresponding quantification of bioluminescence imaging signal (total flux) in the fat pad area. **I** Gross morphology of liver showing metastases on day 20. **J** bioluminescence imaging of lungs on day 20. **K** Immunofluorescence staining of α-SMA (green), SPRY2 (red) and DAPI (blue) in primary breast tumor (upper panel) and lung metastases (lower panel). *P* values based on unpaired Student’s *t* test (**B**, **C** and **G**) or Mann–Whitney test (**D**, **H**): *P < 0.05, **P < 0.01, ***P < 0.001

We next test whether SPRY2 downregulation in the TME controls tumor progression. SPRY2 expression was stably silenced with shRNAs in normal primary mammary gland fibroblasts isolated from mice (Fig. 3A). 4T1 tumor cells were treated with conditioned media (CM) collected from fibroblasts. CCK8 results showed tumor cells proliferation was increased after being treated with CM from sh*Spry2* fibroblasts compared with shNC fibroblasts (Fig. 3B). Consistently, colony formation assay showed increased cell proliferation of tumor cells after co-cultured with sh*Spry2* compared with shNC fibroblasts (Fig. 3C). We then used a direct-contact co-culture model to observe the effect of fibroblast-expressing SPRY2 on tumor cell growth. We co-cultured luciferase labeled 4T1 tumor cells with a monolayer of fibroblasts to quantify tumor cell proliferation by luciferase assays. We found tumor cell numbers increased when co-cultured with Sh*Spry2* fibroblasts compared with control fibroblasts (Additional file 3: Figure S2A).

To investigate the in vivo consequence of SPRY2 knockdown in the TME, we co-injected tumor cells with shNC or sh*Spry2* stromal cells subcutaneously and into tail vein. Consistent with our findings in vitro, SPRY2 knockdown in fibroblasts significantly increased the tumor burden of breast cancer in subcutaneous xenograft model (Fig. 3D). HE, Masson staining and Ki-67 IHC staining showed more matrix deposition and tumor proliferation after SPRY2 knockdown (Fig. 3E). Moreover, we noticed that knockdown of SPRY2 also enhanced in vivo lung metastasis of breast cancer cells (Fig. 3F, G). We then performed direct transplanting of a mixture of luciferase tagged 4T1 cancer cells with shNC or sh*Spry2* fibroblasts into the mammary fat pad of female nude mice. Breast tumor growth was monitored by whole-body bioluminescence imaging at day7, 14 and 20. Tumor growth in mammary fat pad was increased when co-injected with sh*Spry2 f*ibroblasts compared with control fibroblasts (Fig. 3H). Tumor metastases in resected liver and lung were all increased when co-injected with sh*Spry2 f*ibroblasts compared with control fibroblasts (Fig. 3I, J). Immunofluorescence staining of the activated CAF marker a-SMA and SPRY2 in primary tumors and lung metastatic lesions showed decreased SPRY2 expression in a-SMA positive fibroblasts in sh*Spry2* fibroblasts co-injected group compared with shNC group (Fig. 3K). Therefore, these data indicated a cancer-suppressive role of SPRY2 in the TME.

### Loss of SPRY2 promotes fibroblast activation and glycolysis which contribute to fibroblast activation

**Fig. 4:**
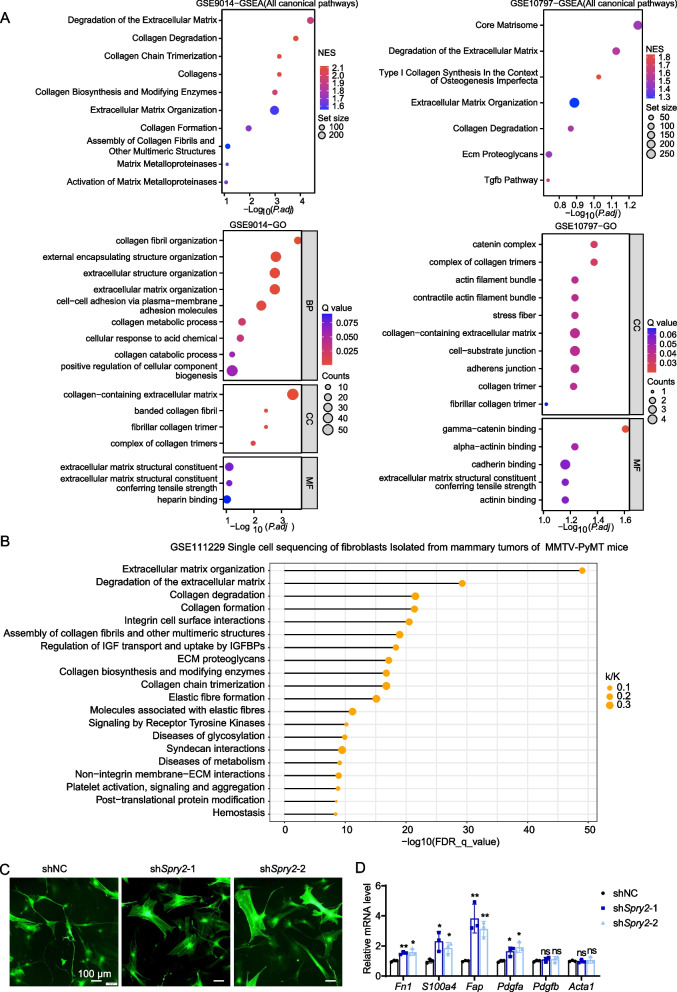
SPRY2 knockdown promotes fibroblast activation in breast cancer stroma. **A** GSEA and GO analysis of increased expression genes comparing *SPRY2*-low group and *SPRY2*-high group by using the RNA sequencing (RNA-seq) data of CAFs from breast cancers in GSE 10797 and GSE9014. **B** Reactome analysis of *Spry2*-low group and *Spry2*-high group by using single-cell-sequencing data of fibroblasts isolated from mammary tumor of MMTV-PyMT mice (GSE111229). **C** Morphologic changes of fibroblasts after transfected with shRNA targeting SPRY2 or scramble with GFP expressing. Scale bar:100 µm. **D** mRNA expression of fibroblast activation markers after SPRY2 knockdown. *P* values based on unpaired Student’s *t* test: *P < 0.05, **P < 0.01

To elucidate the biological functions of SPRY2 in stroma, we first compared the gene expression profiles between *SPRY2*-low group and *SPRY2*-high group using the RNA-seq data of CAFs from breast cancers patients in GSE10797 and GSE9014. GSEA and GO analysis revealed multiple enriched gene sets related to CAFs activation in *SPRY2*-low expression groups in both datasets (Fig. 4A). We then performed Reactome analysis of *Spry2*-low group and *Spry2*-high group by using single-cell-sequencing data of fibroblasts isolated from mammary tumors of MMTV-PyMT mice (GSE111229). Consistently, we found multiple gene sets related with CAF activation in *Spry2*-low group (Fig. 4B). In fibroblasts, SPRY2 knockdown increased cell proliferation (Additional file 3: Figure S2B) and induced morphologic changes where cells exhibited the classical stress fiber structures and the mechanical tensions typical of myofibroblasts (Fig. 4C). mRNA expression of CAFs makers, including *Fn1*, *S100a4*, *Fap* and *Pdgf*α, was increased after SPRY2 knockdown (Fig. 4D). These data indicate loss of SPRY2 enhanced the acquisition of CAF markers and SPRY2 may be a pivotal factor in fibroblast activation.

**Fig. 5:**
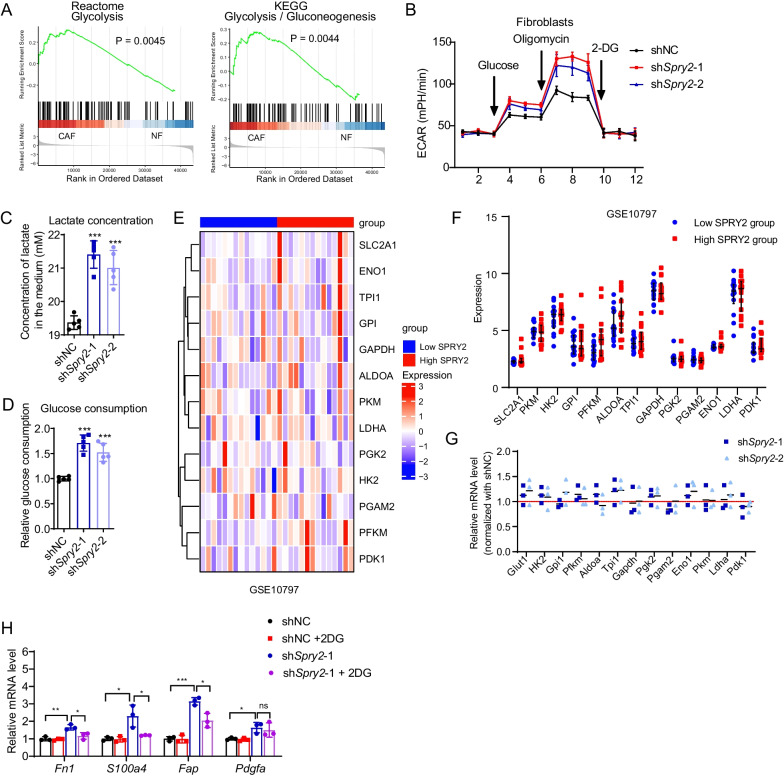
SPRY2 knockdown promotes glycolysis in fibroblasts. **A** GSEA plot based on gene expression in CAFs compared with NFs using the RNA-seq data from GSE 90505. **B** Extracellular acidification rate (ECAR) after SPRY2 knockdown in fibroblasts. **C, D** Lactate production (**C**) and glucose consumption (**D**) after SPRY2 knockdown in fibroblasts. **E**, **F**. Heatmap and graph showing expression of glycolytic enzymes based on the expression of SPRY2 in GSE10797. **G** Relative mRNA expression of glycolytic enzymes after SPRY2 knockdown in fibroblasts (normalized with mRNA expression in shNC group). **H** Relative mRNA expression of fibroblasts activation markers in shNC and sh*Spry2* knockdown fibroblasts with or without 2-DG treatment (normalized with mRNA expression in shNC group). Data are presented as mean ± SD (*n* = 3 independent samples). *P* values based on unpaired Student’s *t* test: *P < 0.05, **P < 0.01, ***P < 0.001

Metabolic reprogramming switched from oxidative phosphorylation (OXPHOS) to glycolysis is found during the conversion of NFs into CAFs [24, 25]. Consistently, by comparing the transcriptional changes in CAFs of breast cancer from GSE90505, we found glycolysis was enriched in CAFs compared with NFs (Fig. 5A). Next, we investigated whether altered SPRY2 levels influence glycolytic activity in mammary fibroblast by measuring extracellular acidification rate (ECAR). Knockdown of SPRY2 significantly increased ECAR levels (Fig. 5B), accompanied by increased lactate production and glucose consumption in fibroblasts (Fig. 5C, D). When we compared the mRNA expression of glycolytic enzymes in SPRY2 low and SPRY2 high samples using datasets from GSE10797, we found no significant difference of glycolytic enzymes between the two groups (Fig. 5E, F). Consistently, mRNA expression of glycolytic enzymes was not changed after SPRY2 knockdown in fibroblasts (Fig. 5G). Interestingly, when cell glycolysis was suppressed with 2-DG, the expression of fibroblast activation markers was decreased (Fig. 5H). The above results suggest a glycolysis-dependent activation by SPRY2 in fibroblasts.

### SPRY2 inhibits LDHA Y10 phosphorylation via interfering with the interaction between SRC and LDHA

**Fig. 6:**
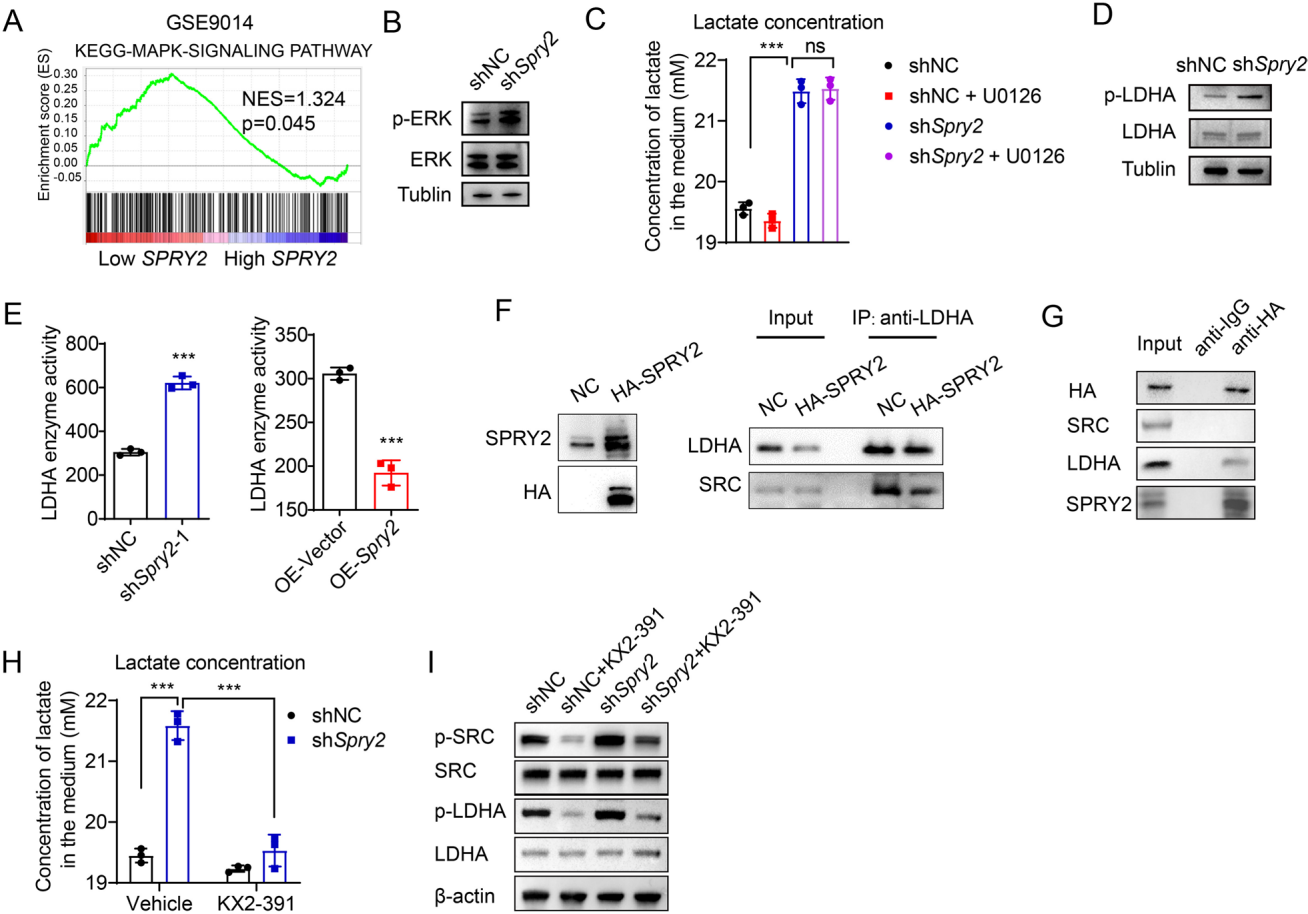
SPRY2 suppresses the Y10 phosphorylation of LDHA by interfering LDHA and SRC interaction. **A** GSEA plot based on the gene expression profiles of *SPRY2*-low group compared with *SPRY2*-high group in GEO data sets GSE9014. NES, normalized enrichment score. False discovery rate (FDR) was set at 0.25. **B** ERK total protein expression and ERK phosphorylation level was determined by western blot after SPRY2 knockdown in fibroblasts. **C** Concentration of lactate in medium after treated with ERK signaling inhibitor U0126 in fibroblasts with SPRY2 knockdown. **D** LDHA total protein expression and LDHA Y10 phosphorylation level was determined by western blot after SPRY2 knockdown in fibroblasts. **E** LDHA enzyme activity in SPRY2 knockdown or overexpressing cells. **F** Co-IP analysis of the interaction between SRC and LDHA in fibroblasts transfected with vector (NC) or HA-Spry2 expressing plasmids. **G** Fibroblasts transfected with HA-Spry2 expressing plasmids were lysed and immunoprecipitated with anti-IgG or anti-HA antibody and immunoblotted with the indicated antibodies. **H** Concentration of lactate in medium of fibroblasts treated with or without SRC inhibitor KX2-391 in fibroblasts with SPRY2 knockdown or not. Data are presented as mean ± SD (*n* = 3 independent samples). **I** Western blot showing protein expression of p-SRC, total SRC, p-LDHA and LDHA in fibroblasts treated with or without SRC inhibitor KX2-391 in fibroblasts with SPRY2 knockdown or not. *P* values based on unpaired Student’s *t* test: *P < 0.05, **P < 0.01, ***P < 0.001

We next aimed to elucidate the molecular mechanism of SPRY2 on glycolysis. GSEA based on SPRY2 expression in GSE9014 showed MAPK signaling pathway was enriched in Low- *SPRY2* expression groups (Fig. 6A). SPRY proteins are evolutionarily conserved modulators of MAPK/ERK pathway; we then examined the activation of MAPK/ERK pathway after SPRY2 knockdown in fibroblasts. We found increased phosphorylation of ERK1/2 after SPRY2 knockdown (Fig. 6B). However, the specific inhibitor targeting MEK1/2, U0126, had no effect on the lactate production after SPRY2 knockdown (Fig. 6C).

Given the reports regarding the regulation of protein phosphorylation by SPRY2, we speculated SPRY2 might regulate glycolytic enzyme activity by phosphorylation modification. LDHA, a critical metabolic enzyme in glycolysis, catalyzes the interconversion of pyruvate and lactate. Since LDHA activity is frequently attributed to its phosphorylation at tyrosine 10, we next examined the effect of SPRY2 on LDHA phosphorylation. By western blot analysis, we found LDHA protein levels were not affected, while phosphorylation at tyrosine 10 was significantly increased after SPRY2 knockdown in fibroblasts (Fig. 6D). Consistently, LDHA enzyme activity was increased after SPRY2 knockdown, whereas SPRY2 overexpression resulted in the opposite effect (Fig. 6E). SPRY2 has been reported to interact with SRC and SRC could phosphorylate LDHA at tyrosine 10 in breast cancer. Therefore, we examined whether SPRY2 affects the interaction between LDHA and SRC. Notably, when SPRY2 was overexpressed, the binding of SRC to LDHA was interfered (Fig. 6F). Co-IP further showed that SPRY2 interacted directly with LDHA, but not SRC in fibroblast (Fig. 6G). SPRY2 knockdown led to a significant increase in lactate production in fibroblasts, which was completely reversed by the SRC kinase inhibitor KX2-391 (Fig. 6H). Consistently, LDHA phosphorylation was found significantly increased after SPRY2 knockdown and was reversed by the SRC kinase inhibitor KX2-391 (Fig. 6I). Taken together, these results suggest that decreased SPRY2 expression in CAFs facilitates metabolic switching toward glycolysis via promoting LDHA phosphorylation by SRC, and enhancing LDHA activity.

In addition, we found glucose starvation significantly suppressed SPRY2 expression in fibroblasts (Additional file 3: Figure S2C), indicating that SPRY2 might be a glucose starvation-suppressed gene and the decreased expression of which could regulate fibroblasts activation via promoting glycolytic metabolism in mammary fibroblasts.

### Loss of SPRY2 in stromal fibroblasts promotes breast cancer stemness dependent on glycolysis

**Fig. 7:**
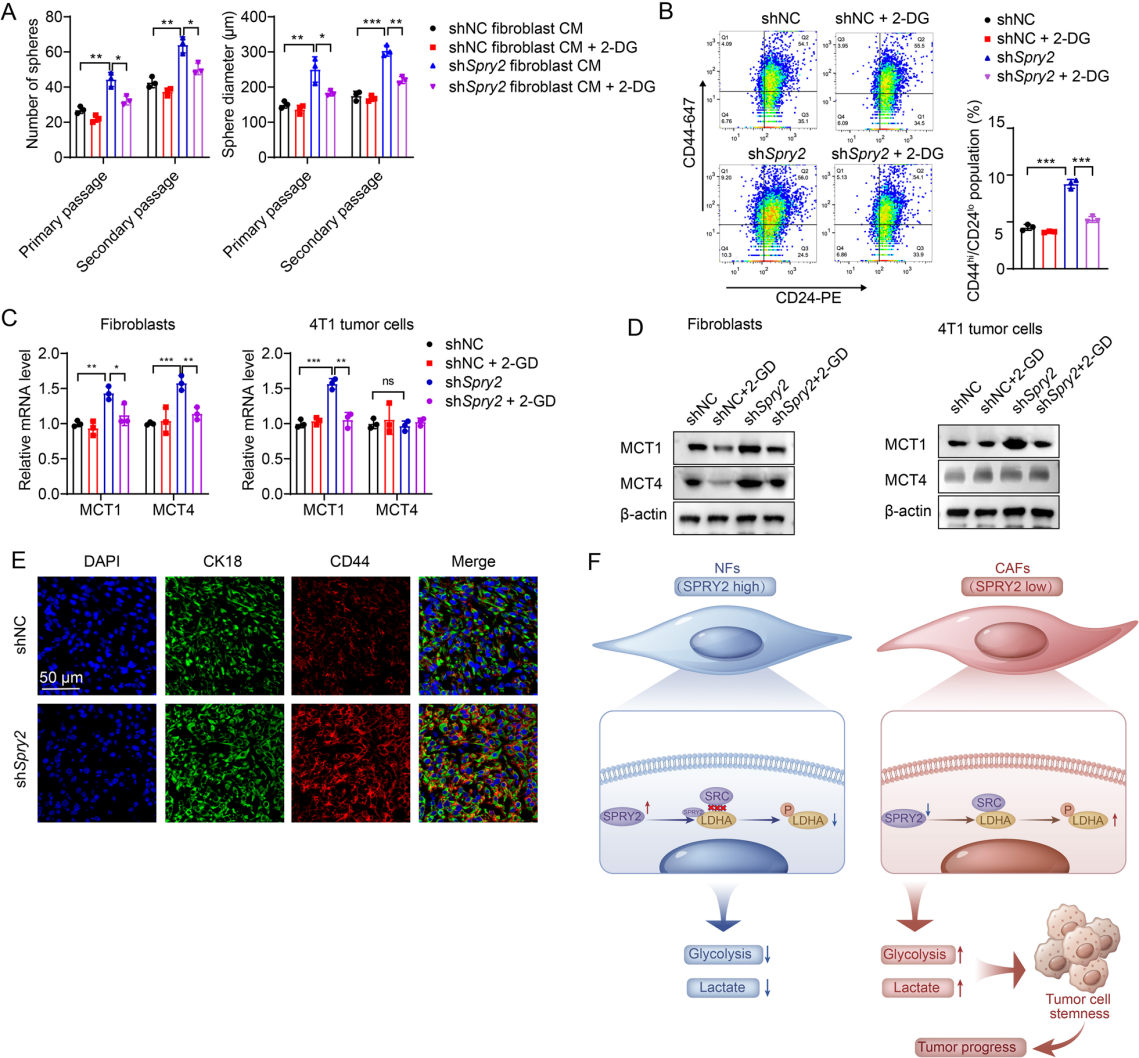
SPRY2 knockdown in stromal fibroblasts promotes breast cancer stemness depending on glycolysis. **A** Sphere establishment in both primary and secondary passages compared with control CM and CM from sh*Spry2* stroma cells pretreated with 2-DG in sphere-formation assays in 4T1 cells. Graph showing sphere formation was increased in 4T1 cells treated with CM from sh*Spry2* stromal cells compared with that treated with control CM. 4T1 cells further formed bigger spheres in diameter under sh*Spry2* stromal cell CM treatment compared with controls. The enhanced sphere-forming abilities of 4T1 cells under treatment of *shSpry2* stromal cell CM were inhibited when treated with CM from sh*Spry2* stroma cells pretreated with 2-DG. Scale bar = 100 μm. **B** Flow cytometry analysis showing the percentage in the CD44^+^ high; CD24^−^ low cell subpopulation in 4T1 cells treated with CM from sh*Spry2* fibroblasts compared to the control and that pretreated with 2-DG. **C** Relative mRNA expression of MCT1 and MCT4 in fibroblasts and 4T1 cells treated with CM from fibroblasts. **D** Protein expression of MCT1 and MCT4 in fibroblasts and 4T1 tumor cells treated with CM from fibroblasts. **E** Immunofluorescence staining of CK18 (green), CD44 (red) and DAPI (blue) in the tumor of mice co-injected with shNC or sh*Spry2* fibroblasts with tumor cells. Scale bar = 50 μm. **F** Model depicting the decreased stromal SPRY2 expression enhance the stem cell ability of breast cancer cells via glycolysis and promotes breast cancer progression. *P* values based on unpaired Student’s *t* test: *P < 0.05, **P < 0.01, ***P < 0.001

Lactate in the microenvironment has been recognized as a key player in regulating the cell stemness. To investigate whether stromal SPRY2 affected breast cancer CSC properties and also clarify whether the effects were dependent on stroma glycolysis, we performed sphere-formation assays. The result showed that treatment of 4T1 cells with CM from sh*Spry2* stromal cells increased sphere establishment in both primary and secondary passages compared with control CM (Fig. 7A). 4T1 cells further formed spheres bigger in diameter under sh*Spry2* stromal cell CM treatment compared with controls (Fig. 7A). Notably, the enhanced sphere-forming abilities of 4T1 cells under treatment of sh*Spry2* stromal cell CM were inhibited when treated with CM from sh*Spry2* stroma cells pretreated with 2-DG (Fig. 7A).

The surface expression of CD44^+^ high; CD24^−^ low cells has been considered a stem population marker of breast cancers. Flow cytometry analysis demonstrated a significant increase in the CD44^+^ high; CD24^−^ low cell subpopulation in 4T1 cells treated with CM from sh*Spry2* fibroblasts compared to the control, while the number was decreased when treated with CM from sh*Spry2* stroma cells pretreated with 2-DG (Fig. 7B). Monocarboxylate transporter MCT1 (SLC16A1) is responsible for the influx of lactate and MCT4 (SLC16A3) is responsible for the efflux of lactate in cells. The mRNA and protein expression of both MCT1 and MCT4 were increased in fibroblasts after SPRY2 knockdown and decreased when pretreated with 2-DG in sh*Spry2* cells. MCT1 expression was increased in 4T1 cells treated with CM from sh*Spry2* fibroblasts and decreased when treated with fibroblasts CM pretreated with 2-DG (Fig. 7C, D).

IHC staining of CD44 in tumors from mice co-injected with tumor cells and shNC or sh*Spry2* fibroblasts showed enhanced CD44 expression in sh*Spry2* groups (Additional file 4: Figure S3A). Further immunofluorescence staining of α-SMA or CK18 with CD44 confirmed the increased CD44 expression in CK18 positive tumor cells (Additional file 4: Figure S3B and Fig. 7E). These results suggest that SPRY2 knockdown in fibroblasts may enhance the stem cell ability of breast cancer cells via glycolysis.

## Discussion

Tumor cell-CAFs cross-talk plays important role in cancer development. As one of the major components of TME, CAFs have been extensively investigated in breast cancer, which is abundant with ECM and stroma cells. In this study, we identified SPRY2 as a novel regulator of CAFs in breast cancer development. Decreased SPRY2 expression in stroma of breast cancer predicts a poor prognosis and contributes to tumor progression (Fig. 7F).

Changes in the self-expression of certain components, for example, Yes-associated protein 1 have been found accompanied during the transformation of NFs into CAFs [26]. Here, we identified another gene SPRY2, whose decreased expression promotes CAFs phenotypes in breast cancer. To the best of our knowledge, the present study is the first to report SPRY2 in regulating CAFs function. Since decreased expression of tumor SPRY2 has been considered to be a significant independent prognostic factor and plays a cancer-promoting role in breast cancer [27], targeting SPRY2 might be a promising approach to achieve therapeutic effects in combating breast cancer. It would also be interesting to pursue whether and how SPRY2 in different cellular compartments could synergize to influence disease progression in future studies.

Our work demonstrated SPRY2 knockdown in breast fibroblasts led to increased glycolysis which further promotes CAFs phenotype. Inactivation of SPRY2 has been reported to activate MAPK and PKM2 pathways and accelerate liver cancer development [28]. Differently from the molecular mechanism of SPRY2 on glycolysis in liver cancer, SPRY2 inhibits glycolysis in a MAPK-independent manner in breast cancer CAFs. As an evolutionarily conserved antagonist of RTK signaling pathways, SPRY2 has been reported to interact with an increasing number of effectors, mediators, and regulators, including SRC, and lead to ultimate influence on RTK signaling activation, [29, 30]. Here, we have identified the interaction between LDHA and SRC, and the phosphorylation of LDHA by SRC was interfered by SPRY2 in mammary fibroblasts. Post-translational modifications, including phosphorylation and acetylation, have been documented as potential regulators of LDHA activity [31, 32]. Phosphorylation of LDHA, at tyrosine 10 and 83 has been found to significantly enhance LDHA tetramer formation and cofactor binding, resulting in increased lactate dehydrogenase activity [14]. Phosphorylation at tyrosine 10 by upstream kinases, HER2 and SRC has been reported to induce activation of LDHA promoting cancer cell invasion, anoikis resistance, and tumor metastasis [31]. As shown by our work, targeting tyrosine phosphorylation of LDHA by SPRY2 might be prospective in stroma of breast cancer.

Our findings elucidated that lactate production by SPRY2 knockdown in stromal fibroblasts promotes tumor cell stemness. For centuries, lactate was considered as the result of tissue hypoxia and a waste product of glucose metabolism. As metabolism has emerged as a key player for stem cell behavior, the role of lactate in regulating the cell stemness has been revelated in recent years [33–36]. Lactate has been found to modulate gene expression in human mesenchymal stem cells and expands the transcriptional network in mouse embryonic stem cells [37, 38]. Human-induced pluripotent stem cells, with metabolic flexibility, could metabolize lactate to support exponential growth [39]. In periodontal ligament stem cells, lactate inhibits osteogenic differentiation through the MCT1-mTOR signaling pathway [40]. The work of *Wang *et al*.* published in *Cell Stem Cell* show that brain endothelial cells directly affect adult neural stem cells by regulating lactate levels, which provided strong evidence for the effect of lactate in the microenvironment on stem cell activity [41, 42]. Consistent with its role on stem cells, recent work showed lactate could be used as an energy source by the cancer cells and recognized as a mediator in a cell–cell cross-talk in cancers [43, 44]. However, lactate might show pleotropic effects as promoter and inhibitor of tumorigenesis depending on context, as besides tumor cells, lactate also affect the work of immune cells. Lactate can overtake glucose as a primary carbon fuel source for a majority of tissues including immune organs [45, 46]. Lactate can augment the anti-tumor immunity of CD8^ +^ T cells. CD8^+^ T cells pretreated with lactate efficiently inhibit tumor growth upon adoptive transfer to tumor-bearing mice, providing evidence for the anti-tumor immunity role of lactate independent of pH-dependent effect [36]. In breast cancer, lactate activates macrophage Gpr132 to transform into the M2-like phenotype, uncovering the lactate-Gpr132 axis as a driver of breast cancer metastasis by stimulating tumor-macrophage interplay [47]. Here, our study indicates the role of lactate in mediating stromal fibroblast-tumor cell interaction in breast cancers. However, the effect of lactate on cancer might be highly complex and hard to completely decipher in the microenvironment, the molecular bases of lactate on tumor cell stemness remain further investigation.

In conclusion, our study showed that dysregulated SPRY2 in TME could promote breast tumor development. Mechanistically, we uncovered SPRY2 interfered with SRC-LDHA interaction to inhibit LDHA phosphorylation and suppress glycolysis in tumor stroma. These findings demonstrate that point towards SPRY2-glycolysis in CAFs a potential therapeutic target in breast cancer.

## Supplementary Information

**Additional file 1. Table S1.** Sequence of primers in qRT-PCR

**Additional file 2. Figure S1.** SPRYs and SPREDS expression in breast cancers. Single cell sequencing analysis showing the expression of SPRY1, SPRY2, SPRY3, SPRY4, SPRED1, SPRED2 and SPRED3 in different cell types of human breast cancers. Analyzed with scRNA-seq datasets using online tool TISCH2 (Tumor Immune Single-cell Hub 2, http://tisch.comp-genomics.org/home/).

**Additional file 3. Figure S2.** **A**. Quantification of relative luciferase-tagged 4T1 cells proliferation after cocultured with control or sh*Spry2* fibroblasts for 4 days by luciferase assays (n= 3). **p<0.01. **B**. Relative cell proliferation of fibroblasts 4 days after Spry2 knockdown showing by CCK8 assay (n= 3). *p<0.05, **p<0.01. **C**. Relative Spry2 mRNA expression in fibroblasts cultured in medium with 10% and 0% serum glucose for 24 h. **p < 0.01.

**Additional file 4. Figure S3.** **A**. IHC staining of CD44 in the tumor of mice co-injected with shNC or sh*Spry2* fibroblasts with 4T1 cells. Scale bar = 50 μm. **B**. Immunofluorescence staining of α-SMA (green), CD44 (red) and DAPI (blue) in the tumor of mice co-injected with shNC or sh*Spry2* fibroblasts with 4T1 cells. Scale bar = 50 μm.

## Data Availability

The data are available within the Article, Supplementary Information, or available from the authors upon request. Source data are provided with this paper.
