# Supplementary material for: Loss of SPRY2 contributes to cancer-associated fibroblasts activation and promotes breast cancer development
Source: Breast Cancer Res. 2023 Jul 28;25:90. doi: 10.1186/s13058-023-01683-8 (PMC10375677; doi:10.1186/s13058-023-01683-8)
Supplement: Supplementary file 1 — Additional file 1. Table S1. Sequence of primers in qRT-PCR [file 13058_2023_1683_MOESM1_ESM.docx]

**Supplementary table 1**. **Sequence of primers in qRT-PCR**

| Gene | Primer Sequence (5’-3’) | |
| --- | --- | --- |
|  | Forward | Reverse |
| *Spry2* | TCCAAGAGATGCCCTTACCCA | GCAGACCGTGGAGTCTTTCA |
| *Fn1* | ATGTGGACCCCTCCTGATAGT | GCCCAGTGATTTCAGCAAAGG |
| *S100a4* | TCCACAAATACTCAGGCAAAGAG | GCAGCTCCCTGGTCAGTAG |
| *Fap* | GTCACCTGATCGGCAATTTGT | CCCCATTCTGAAGGTCGTAGAT |
| *Pdgfa* | GAGGAAGCCGAGATACCCC | TGCTGTGGATCTGACTTCGAG |
| *Pdgfb* | CATCCGCTCCTTTGATGATCTT | GTGCTCGGGTCATGTTCAAGT |
| *Acta1* | CCCAAAGCTAACCGGGAGAAG | CCAGAATCCAACACGATGCC |
| *Glut1* | CAGTTCGGCTATAACACTGGTG | GCCCCCGACAGAGAAGATG |
| *Hk1* | AGGGCGCATTACTCCAGAG | CCCTGTGGGTGTCTTGTGTG |
| *Gpi1* | TCAAGCTGCGCGAACTTTTTG | GGTTCTTGGAGTAGTCCACCAG |
| *Pfkm* | TGTGGTCCGAGTTGGTATCTT | GCACTTCCAATCACTGTGCC |
| *Aldoa* | CGTGTGAATCCCTGCATTGG | CAGCCCCTGGGTAGTTGTC |
| *Tpi1* | CCAGGAAGTTCTTCGTTGGGG | CAAAGTCGATGTAAGCGGTGG |
| *Gapdh* | AGGTCGGTGTGAACGGATTTG | TGTAGACCATGTAGTTGAGGTCA |
| *Pgk2* | TTCTGCTAAGTTGACTCTGGACA | AGCCTTGATTCTCTGGTTGTTTG |
| *Pgam2* | TGGAACCAAGAGAACCGTTTC | TGGCATCTTTGATAGCGGTGG |
| *Eno1* | TGCGTCCACTGGCATCTAC | CAGAGCAGGCGCAATAGTTTTA |
| *Pkm* | GCCGCCTGGACATTGACTC | CCATGAGAGAAATTCAGCCGAG |
| *Ldha* | TGTCTCCAGCAAAGACTACTGT | GACTGTACTTGACAATGTTGGGA |
| *Pdk1* | GGACTTCGGGTCAGTGAATGC | TCCTGAGAAGATTGTCGGGGA |
| *MCT1* | TGTTAGTCGGAGCCTTCATTTC | CACTGGTCGTTGCACTGAATA |
| *MCT4* | TCACGGGTTTCTCCTACGC | GCCAAAGCGGTTCACACAC |
