# Supplementary material for: Loss of SPRY2 contributes to cancer-associated fibroblasts activation and promotes breast cancer development
Source: Breast Cancer Res. 2023 Jul 28;25:90. doi: 10.1186/s13058-023-01683-8 (PMC10375677; doi:10.1186/s13058-023-01683-8)
Supplement: Supplementary file 2 — Additional file 2. Figure S1. SPRYs and SPREDS expression in breast cancers. Single cell sequencing analysis showing the expression of SPRY1, SPRY2, SPRY3, SPRY4, SPRED1, SPRED2 and SPRED3 in different cell types of human breast cancers. Analyzed with scRNA-seq datasets using online tool TISCH2 (Tumor Immune Single-cell Hub 2, http://tisch.comp-genomics.org/home/). [file 13058_2023_1683_MOESM2_ESM.docx]

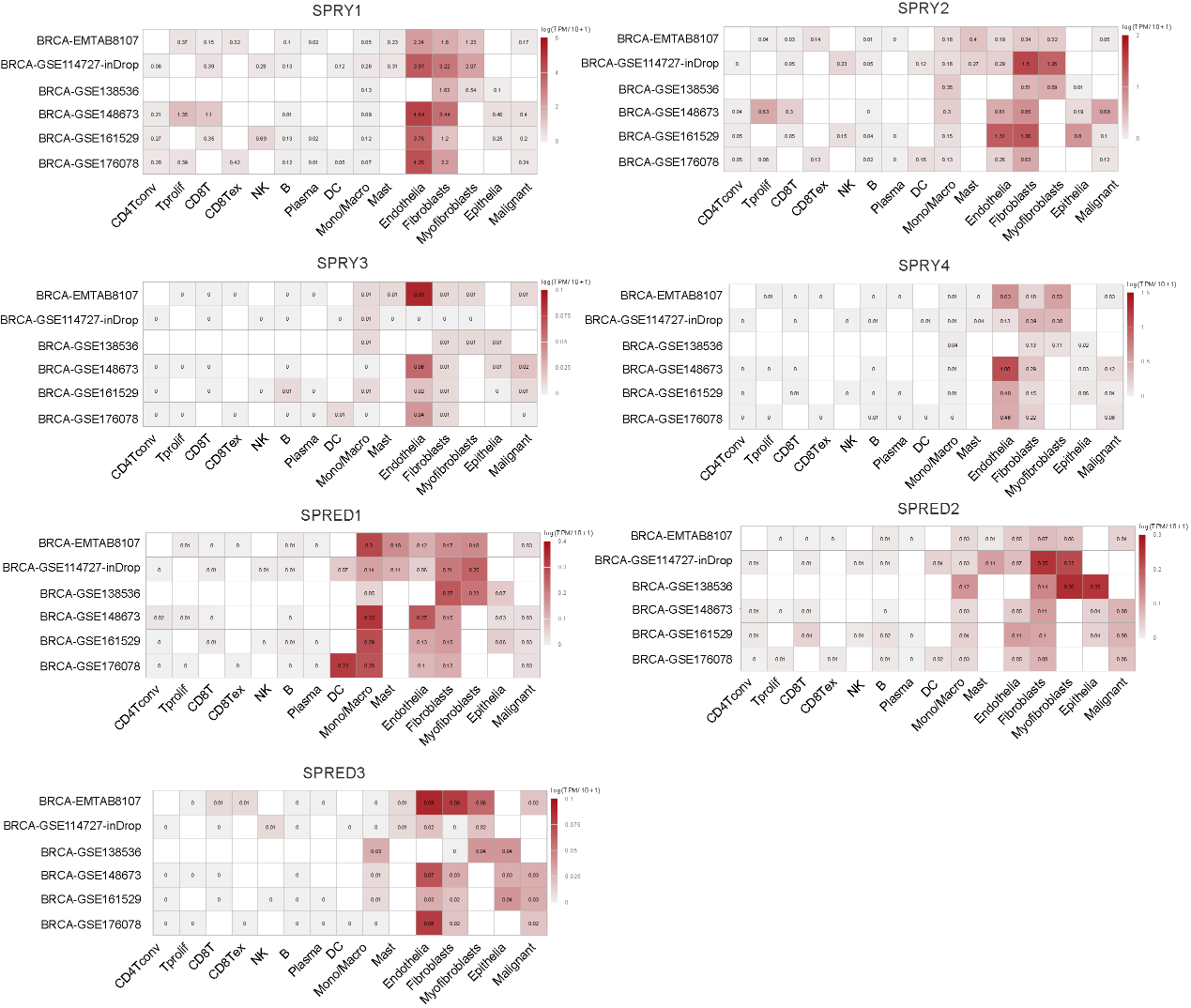
**Supplementary figure 1. SPRYs and SPREDS expression in breast cancers.** Single cell sequencing analysis showing the expression of SPRY1, SPRY2, SPRY3, SPRY4, SPRED1, SPRED2 and SPRED3 in different cell types of human breast cancers. Analyzed with scRNA-seq datasets using online tool TISCH2 (Tumor Immune Single-cell Hub 2, [*http://tisch.comp-genomics.org/home/*](http://tisch.comp-genomics.org/home/)).
