# Supplementary material for: Loss of SPRY2 contributes to cancer-associated fibroblasts activation and promotes breast cancer development
Source: Breast Cancer Res. 2023 Jul 28;25:90. doi: 10.1186/s13058-023-01683-8 (PMC10375677; doi:10.1186/s13058-023-01683-8)
Supplement: Supplementary file 3 — Additional file 3. Figure S2. A. Quantification of relative luciferase-tagged 4T1 cells proliferation after cocultured with control or shSpry2 fibroblasts for 4 days by luciferase assays (n= 3). **p<0.01. B. Relative cell proliferation of fibroblasts 4 days after Spry2 knockdown showing by CCK8 assay (n= 3). *p<0.05, **p<0.01. C. Relative Spry2 mRNA expression in fibroblasts cultured in medium with 10% and 0% serum glucose for 24 h. **p < 0.01. [file 13058_2023_1683_MOESM3_ESM.docx]

**
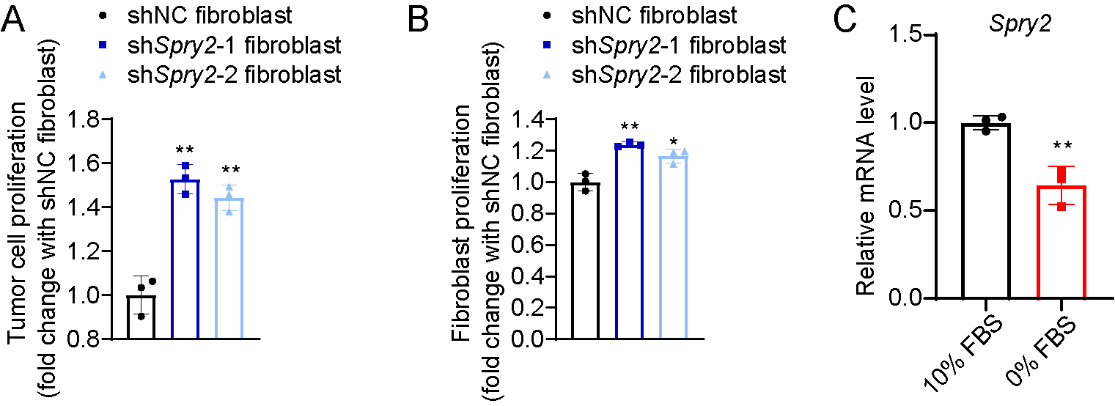
**

**Supplementary figure 2.** A. Quantification of relative luciferase-tagged 4T1 cells proliferation after cocultured with control or sh*Spry2* fibroblasts for 4 days by luciferase assays (n= 3). **p<0.01. B. Relative cell proliferation of fibroblasts 4 days after Spry2 knockdown showing by CCK8 assay (n= 3). *p<0.05, **p<0.01. C. Relative *Spry2* mRNA expression in fibroblasts cultured in medium with 10% and 0% serum glucose for 24 h. **p < 0.01.
