# Supplementary material for: Loss of SPRY2 contributes to cancer-associated fibroblasts activation and promotes breast cancer development
Source: Breast Cancer Res. 2023 Jul 28;25:90. doi: 10.1186/s13058-023-01683-8 (PMC10375677; doi:10.1186/s13058-023-01683-8)
Supplement: Supplementary file 4 — Additional file 4. Figure S3. A. IHC staining of CD44 in the tumor of mice co-injected with shNC or shSpry2 fibroblasts with 4T1 cells. Scale bar = 50 μm. B. Immunofluorescence staining of α-SMA (green), CD44 (red) and DAPI (blue) in the tumor of mice co-injected with shNC or shSpry2 fibroblasts with 4T1 cells. Scale bar = 50 μm. [file 13058_2023_1683_MOESM4_ESM.docx]

**
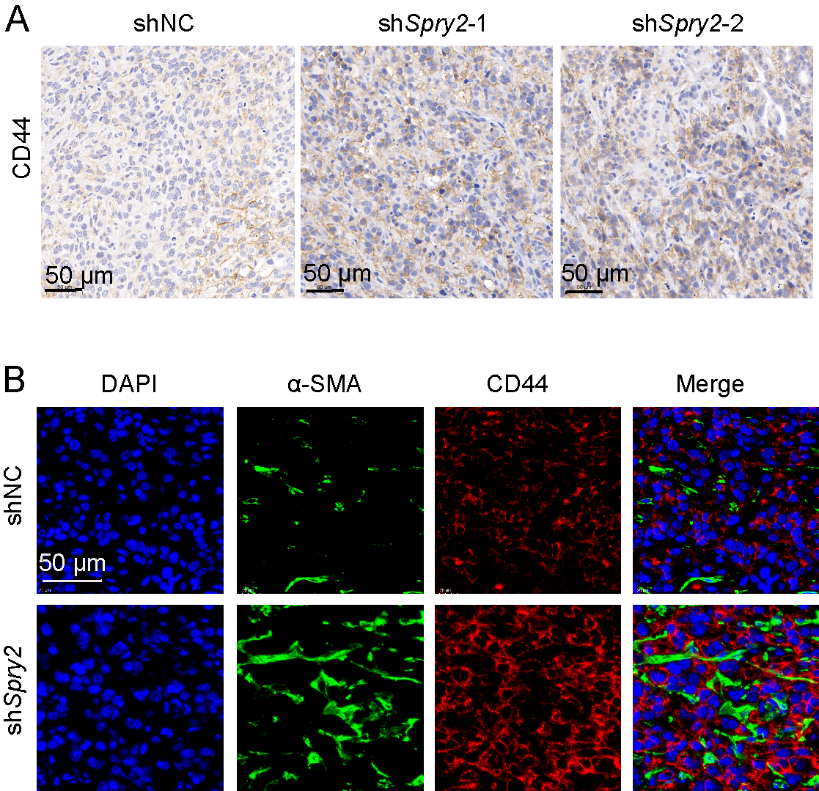
**

**Supplementary figure 3.** A. IHC staining of CD44 in the tumor of mice co-injected with shNC or sh*Spry2* fibroblasts with 4T1 cells. Scale bar = 50 μm. B. Immunofluorescence staining of α-SMA (green), CD44 (red) and DAPI (blue) in the tumor of mice co-injected with shNC or sh*Spry2* fibroblasts with 4T1 cells. Scale bar = 50 μm.
